# Supplementary material for: Pyruvate Dehydrogenase Kinase 1 inhibition mediated oxidative phosphorylation enhancement in cartilage promotes osteoarthritis progression
Source: BMC Musculoskelet Disord. 2023 Jul 20;24:597. doi: 10.1186/s12891-023-06585-6 (PMC10357736; doi:10.1186/s12891-023-06585-6)
Supplement: Supplementary file 1 — Additional file 1. [file 12891_2023_6585_MOESM1_ESM.zip › BP.Gsea.1653623667859/GOBP_POSITIVE_REGULATION_OF_TOR_SIGNALING.html]

Details for gene set GOBP\_POSITIVE\_REGULATION\_OF\_TOR\_SIGNALING[GSEA]

|  || Dataset | 55457\_collapsed\_to\_symbols.cls.cls #OA\_versus\_normal.cls.cls #OA\_versus\_normal\_repos |
| Phenotype | cls.cls#OA\_versus\_normal\_repos |
| Upregulated in class | OA |
| GeneSet | GOBP\_POSITIVE\_REGULATION\_OF\_TOR\_SIGNALING |
| Enrichment Score (ES) | 0.38228905 |
| Normalized Enrichment Score (NES) | 1.4356996 |
| Nominal p-value | 0.055023924 |
| FDR q-value | 0.4502761 |
| FWER p-Value | 1.0 |
Table: GSEA Results Summary

  

Fig 1: Enrichment plot: GOBP\_POSITIVE\_REGULATION\_OF\_TOR\_SIGNALING      
 Profile of the Running ES Score & Positions of GeneSet Members on the Rank Ordered List

  

| SYMBOL | TITLE | RANK IN GENE LIST | RANK METRIC SCORE | RUNNING ES | CORE ENRICHMENT || 1 | LAMTOR5 | "late endosomal/lysosomal adaptor, MAPK and MTOR activator 5 [Source:HGNC Symbol;Acc:HGNC:17955]" | 187 | 0.829 | 0.0544 | Yes |
| 2 | TREM2 | triggering receptor expressed on myeloid cells 2 [Source:HGNC Symbol;Acc:HGNC:17761] | 342 | 0.726 | 0.1029 | Yes |
| 3 | SEC13 | "SEC13 homolog, nuclear pore and COPII coat complex component [Source:HGNC Symbol;Acc:HGNC:10697]" | 460 | 0.677 | 0.1501 | Yes |
| 4 | RRAGA | Ras related GTP binding A [Source:HGNC Symbol;Acc:HGNC:16963] | 530 | 0.647 | 0.1986 | Yes |
| 5 | RRAGB | Ras related GTP binding B [Source:HGNC Symbol;Acc:HGNC:19901] | 953 | 0.528 | 0.2097 | Yes |
| 6 | RRAGD | Ras related GTP binding D [Source:HGNC Symbol;Acc:HGNC:19903] | 1186 | 0.479 | 0.2315 | Yes |
| 7 | LAMTOR2 | "late endosomal/lysosomal adaptor, MAPK and MTOR activator 2 [Source:HGNC Symbol;Acc:HGNC:29796]" | 1283 | 0.461 | 0.2624 | Yes |
| 8 | HDAC3 | histone deacetylase 3 [Source:HGNC Symbol;Acc:HGNC:4854] | 1325 | 0.454 | 0.2970 | Yes |
| 9 | GAS6 | growth arrest specific 6 [Source:HGNC Symbol;Acc:HGNC:4168] | 1942 | 0.368 | 0.2797 | Yes |
| 10 | KLHL22 | kelch like family member 22 [Source:HGNC Symbol;Acc:HGNC:25888] | 1982 | 0.363 | 0.3069 | Yes |
| 11 | GPR137B | G protein-coupled receptor 137B [Source:HGNC Symbol;Acc:HGNC:11862] | 2065 | 0.355 | 0.3300 | Yes |
| 12 | MLST8 | "MTOR associated protein, LST8 homolog [Source:HGNC Symbol;Acc:HGNC:24825]" | 2259 | 0.331 | 0.3426 | Yes |
| 13 | NCKAP1L | NCK associated protein 1 like [Source:HGNC Symbol;Acc:HGNC:4862] | 2560 | 0.296 | 0.3439 | Yes |
| 14 | PIH1D1 | PIH1 domain containing 1 [Source:HGNC Symbol;Acc:HGNC:26075] | 2667 | 0.283 | 0.3592 | Yes |
| 15 | LAMTOR3 | "late endosomal/lysosomal adaptor, MAPK and MTOR activator 3 [Source:HGNC Symbol;Acc:HGNC:15606]" | 2674 | 0.283 | 0.3823 | Yes |
| 16 | LIN28A | lin-28 homolog A [Source:HGNC Symbol;Acc:HGNC:15986] | 3140 | 0.236 | 0.3658 | No |
| 17 | CTNS | "cystinosin, lysosomal cystine transporter [Source:HGNC Symbol;Acc:HGNC:2518]" | 3337 | 0.218 | 0.3687 | No |
| 18 | TELO2 | telomere maintenance 2 [Source:HGNC Symbol;Acc:HGNC:29099] | 3650 | 0.190 | 0.3603 | No |
| 19 | GOLPH3 | golgi phosphoprotein 3 [Source:HGNC Symbol;Acc:HGNC:15452] | 4420 | 0.119 | 0.3104 | No |
| 20 | SIK3 | SIK family kinase 3 [Source:HGNC Symbol;Acc:HGNC:29165] | 5476 | 0.035 | 0.2313 | No |
| 21 | RELN | reelin [Source:HGNC Symbol;Acc:HGNC:9957] | 5635 | 0.021 | 0.2208 | No |
| 22 | MIOS | meiosis regulator for oocyte development [Source:HGNC Symbol;Acc:HGNC:21905] | 5858 | 0.005 | 0.2040 | No |
| 23 | RRAGC | Ras related GTP binding C [Source:HGNC Symbol;Acc:HGNC:19902] | 6202 | -0.021 | 0.1790 | No |
| 24 | PIK3CA | "phosphatidylinositol-4,5-bisphosphate 3-kinase catalytic subunit alpha [Source:HGNC Symbol;Acc:HGNC:8975]" | 7733 | -0.136 | 0.0714 | No |
| 25 | AKT3 | AKT serine/threonine kinase 3 [Source:HGNC Symbol;Acc:HGNC:393] | 8008 | -0.156 | 0.0631 | No |
| 26 | LEP | leptin [Source:HGNC Symbol;Acc:HGNC:6553] | 8740 | -0.212 | 0.0239 | No |
| 27 | FLCN | folliculin [Source:HGNC Symbol;Acc:HGNC:27310] | 8848 | -0.221 | 0.0340 | No |
| 28 | CLEC16A | C-type lectin domain containing 16A [Source:HGNC Symbol;Acc:HGNC:29013] | 10013 | -0.323 | -0.0296 | No |
| 29 | WAC | WW domain containing adaptor with coiled-coil [Source:HGNC Symbol;Acc:HGNC:17327] | 10514 | -0.375 | -0.0373 | No |
| 30 | SEH1L | SEH1 like nucleoporin [Source:HGNC Symbol;Acc:HGNC:30379] | 10651 | -0.388 | -0.0156 | No |
| 31 | HTR6 | 5-hydroxytryptamine receptor 6 [Source:HGNC Symbol;Acc:HGNC:5301] | 11034 | -0.435 | -0.0091 | No |
| 32 | GPR137 | G protein-coupled receptor 137 [Source:HGNC Symbol;Acc:HGNC:24300] | 11734 | -0.539 | -0.0186 | No |
| 33 | WDR59 | WD repeat domain 59 [Source:HGNC Symbol;Acc:HGNC:25706] | 12025 | -0.599 | 0.0087 | No |
| 34 | LARS1 | leucyl-tRNA synthetase 1 [Source:HGNC Symbol;Acc:HGNC:6512] | 12415 | -0.712 | 0.0377 | No |
Table: GSEA details [plain text format]

  

Fig 2: GOBP\_POSITIVE\_REGULATION\_OF\_TOR\_SIGNALING      
 Blue-Pink O' Gram in the Space of the Analyzed GeneSet

  

Fig 3: GOBP\_POSITIVE\_REGULATION\_OF\_TOR\_SIGNALING: Random ES distribution      
 Gene set null distribution of ES for **GOBP\_POSITIVE\_REGULATION\_OF\_TOR\_SIGNALING**
